# Earned Media and Public Engagement With CDC’s "Tips From Former Smokers" Campaign: An Analysis of Online News and Blog Coverage

**DOI:** 10.2196/jmir.3645

**Published:** 2015-01-20

**Authors:** Rachel Kornfield, Katherine Clegg Smith, Glen Szczypka, Lisa Vera, Sherry Emery

**Affiliations:** ^1^School of Journalism and Mass CommunicationUniversity of Wisconsin-MadisonMadison, WIUnited States; ^2^Johns Hopkins Bloomberg School of Public HealthDepartment of Health, Behavior and SocietyBaltimore, MDUnited States; ^3^Institute for Health Research and PolicyUniversity of Illinois at ChicagoChicago, ILUnited States; ^4^Moores Cancer CenterUniversity of California San DiegoLa Jolla, CAUnited States

**Keywords:** mass media, tobacco, health communication

## Abstract

**Background:**

In March 2012, the US Centers for Disease Control and Prevention (CDC) launched the first-ever paid national tobacco education campaign. At a cost of US $54 million, “Tips from Former Smokers” (Tips) ran for 3 months across multiple media, depicting the suffering experienced by smokers and their families in graphic detail. The potential impact and reach of the Tips campaign was not limited to that achieved through paid media placements. It was also potentially extended through “earned media”, including news and blog coverage of the campaign. Such coverage can shape public understanding of and facilitate public engagement with key health issues.

**Objective:**

To better understand the contribution of earned media to the public’s engagement with health issues in the current news media environment, we examined the online “earned media” and public engagement generated by one national public health campaign.

**Methods:**

We constructed a purposive sample of online media coverage of the CDC’s 2012 Tips from Former Smokers television campaign, focusing on 14 influential and politically diverse US news outlets and policy-focused blogs. We identified relevant content by combining campaign and website-specific keywords for 4 months around the campaign release. Each story was coded for content, inclusion of multimedia, and measures of audience engagement.

**Results:**

The search yielded 36 stories mentioning Tips, of which 27 were focused on the campaign. Story content between pieces was strikingly similar, with most stories highlighting the same points about the campaign’s content, cost, and potential impact. We saw notable evidence of audience engagement; stories focused on Tips generated 9547 comments, 8891 Facebook “likes”, 1027 tweets, and 505 story URL shares on Facebook. Audience engagement varied by story and site, as did the valence and relevance of associated audience comments. Comments were most oppositional on CNN and most supportive on Yahoo. Comment coding revealed approximately equal levels of opposition and support overall. We identified four common arguments among oppositional comments: government intrusion on personal behaviors, problematic allocation of governmental spending, questionable science, and challenges regarding campaign efficacy. Supportive comments tended to convey personal stories and emotions.

**Conclusions:**

The Tips campaign received limited coverage on either online news or blog sources, but the limited number of stories generated engagement among online audiences. In addition to the content and volume of blog and news coverage, audience comments and websites’ mechanisms for sharing stories via social media are likely to determine the influence of online earned media. In order to facilitate meaningful evaluation of public health campaigns within the rapidly advancing media environment, there is a need for the public health community to build consensus regarding collection and assessment of engagement data.

## Introduction

In March 2012, the US Centers for Disease Control and Prevention (CDC) launched “Tips From Former Smokers” (Tips) [1], the first-ever paid national tobacco education campaign. Tips ran for 3 months across multiple media—television, radio, movie theaters, online, billboards, newspapers, and magazines—at a cost of US $54 million. Featuring graphic portrayals of real people suffering from smoking-related illnesses, the campaign aimed to build awareness of the immediate damaging effects of smoking on smokers as well as those exposed to secondhand smoke and to encourage smokers both to quit and to not smoke around others. Tips’ primary target audience was smokers aged 18-54.

Health communication campaigns can be powerful when well-crafted messages are placed where members of target audiences are likely to see or hear them. Exposure to a campaign such as Tips can be separated into “paid” and “earned” media. Paid media is similar to conventional advertising: a market-based fee is paid for the placement of campaign ads in front of a target audience. Earned media includes donated media that appears very similar to paid media, for example, public service announcements (PSAs), and also includes discussion or coverage of an issue or campaign in the news. To the extent that some aspect of a campaign sparks journalistic interest (among both “formal” journalists and “citizen” journalists or bloggers), earned media may increase exposure to a campaign message without incurring additional cost. Earned media also may spread a message beyond the targeted audience, convey pertinent background information about an issue, and heighten issue salience among stakeholders as well as the general public [2-5]. Both paid and earned media can play a role in a campaign’s reach and potential capacity to bring about desired change.

Recent changes to the journalistic landscape, particularly the emergence and growth of online news [6], provide a platform for earned media to spread with unprecedented speed and potentially well beyond the audience exposed through the paid campaign [7]. The online communication landscape now enables a multidirectional flow of information where consumers increasingly encounter content that is tailored to their interests in a format that facilitates immediate engagement, response, and sharing with one’s social network [8]. For example, a description of a campaign may feature multimedia elements, such as campaign spot videos or interviews with viewers, and such information is likely to appear alongside entertainment and analysis [9]. Further, many news and blog platforms provide opportunities for the public to interact with content by posting public comments, rating or “liking” stories, or sharing content through other social media platforms [10]. The avenues for active audience participation and sharing provided by online outlets may potentially expand the reach and influence of online earned media beyond that possible with traditional earned media.

Simultaneously, the online media environment holds the potential to undermine support for public health campaigns. For example, some have suggested that the proliferation of online sources might in fact have narrowed the type of coverage to which some readers are exposed. Thus, while it is easier than ever to find content to match one’s interests, it is also now quite possible for people to avoid exposure to content that challenges their current opinions and beliefs [11]. It is also potentially more difficult to differentiate between credible and non-credible sources [7,12,13]. Furthermore, several recent experimental studies indicate ways that audience interaction presented alongside online content might undermine campaign messages. For example, in one study, the civility of audience-generated comments significantly influenced readers’ perceptions of a science news story [14]. Similar effects were found in relation to an anti-smoking video PSA: the presence of comments was associated with viewers evaluating the PSA as less effective, particularly when the commentary was negative [15]. The authors proposed that user-submitted commentary, even when supportive, may disrupt the transportive property of a PSA.

The online media environment is also subject to both journalist and audience-generated attempts at message agenda-setting and framing, both of which influence the impact of a health campaign. Agenda-setting refers to placing emphasis on specific issues in a media message in order to influence the importance the audience attributes to that message [16]. Framing may be understood as efforts, either by the communicator of a message or its recipient, to build a context within which a problem is defined, its causes diagnosed, and moral reactions and remedies suggested [17]. Agenda-setting relies on story selection as an indicator of issue importance, while framing focuses on the way those issues are presented and processed [16]. Given the polarized nature of many health policy debates [18], including those around tobacco control [19-23], it becomes important to understand these complex and dynamic interactions. From an agenda-setting and framing perspective, it is important to consider that, in the online setting, not only do journalists set the stage for determining which issues attract audience attention and how that attention is characterized, but audience reaction (such as user-generated comments, likes, and shares) also influences the stories and angles on which journalists choose to focus [24]. Earned media around a campaign such as Tips can affect both the importance the public places on tobacco control and the framework of opinion within which the public views the issue.

In a traditional print news environment, public debates around tobacco control feature a range of narrative, informational, and economic appeals [19,21] as well as arguments regarding the role of the state and individual liberties. While similar appeals likely emerge in online public debates, many online news and opinion (blog) sites also feature distinct norms and standards of public engagement. Notably, online public engagement often features incivility and “trolling”, perhaps due in part to the anonymity that online comment forums afford to those who engage [25-30]. Further, there is substantial variability in levels at which different online platforms implement codes of conduct and curate or monitor comment fields [10]. While some sites discourage or even edit out off-topic or uncivil threads, other platforms allow users to introduce entirely new subjects, with or without explicit reference to the original story. In the context of health policy and media campaigns, online earned media coverage can provide a forum for public engagement with important issues. In contrast, public engagement on online forums can also serve as an avenue for spreading oppositional arguments, or even misinformation, which might serve to undermine these same campaigns’ messages [31]. To date, little is known about how online earned media and the related public debate it generates might affect campaign success.

New methods are required to fully understand the extent, character, and influence of earned media in an online context [7]. Given its unprecedented scale and graphic content, the CDC Tips campaign had the potential to generate considerable earned media, providing new opportunities for the public to encounter and engage with the campaign. We seek to contribute to understanding the role of online earned media by analyzing news coverage about Tips and the level and type of public engagement that it generated. In this paper, we examine news and blogs from prominent online sources during and immediately following the first wave of the campaign in 2012, quantifying the total number of news stories and blog postings and characterizing their content and focus. We also describe total public engagement reported alongside each story, including numbers of shares, likes, and comments garnered. Finally, we characterize the content of a sample of audience-generated comments, including the extent to which these specifically reference the campaign and their valence with regard to tobacco control. We also describe major themes in oppositional and supportive comments.

The digital media landscape and the data it yields offer opportunities to conduct qualitative research on a quantitative scale. Examining the amount of earned coverage about Tips and analyzing the themes and valence of both news stories and responsive comments contributes to the development of effective methods for measuring and analyzing earned media and subsequent public engagement. Such methods will be crucial to building support for future public health campaigns within the rapidly advancing media environment.

## Methods

In October 2012, we constructed a purposive, diverse sample of online news and blog coverage of the CDC’s Tips From Former Smokers television campaign, which aired between March and June 2012. We constructed a politically diverse sample of blog and news sites including five leading US policy-focused blogs (Huffington Post, Politico, Daily Beast, RedState, and the National Review) and nine news media sites (MSNBC, Yahoo News, CNN, Fox News, New York Times, USA Today, Wall Street Journal, Los Angeles Times, Washington Times). News and blog sources were selected based on their readership/audience as well as balance between liberal and conservative perspectives. Traditional news sites were selected from among major regional and national sources to represent the breadth of mainstream political perspectives, and Tips-related keywords were used to search each selected site for relevant articles. Traditional sites also were chosen on the basis of their estimated readership numbers. Audience estimates were collected from Nielsen Insights [32] and from comScore [33]. Readership numbers are presented in Table 1.

**Table 1:** Readership figures for traditional news sources.

News website	Nielsen (May 2012)	comScore (August 2012)
CNN	39,559,000	38,979,000
Daily Beast^a^	16,000,000	16,000,000
Fox News	21,555,000	29,866,000
Huffington Post	29,016,000	43,700,000
LA Times^a^	23,000,000	23,000,000
NY Times	29,248,000	73,099,000
USA Today	15,104,000	26,300,000^a^
MSNBC	30,175,000	123,337,000^a^
Wall Street Journal^a^	13,971,000	13,971,000
Yahoo	142,959,000	163,723,000
Washington Times	9,750,000	9,750,000

^a^Numbers retrieved from the website’s media kit.

Constructing a purposive sample of relevant, influential blogs was somewhat more challenging as we are unaware of any publicly available definitive source of data on blog reach. We therefore referenced various blog ranking sites (eg, technocrati, bynd, ebizmba) to construct our sample by identifying examples of searchable blogs representing diverse perspectives on policy initiatives. We used Google and Google Advanced search engines to conduct our searches, combining website-specific keywords (eg, “CNN”) with a series of keyword combinations related to Tips. We collected all media pieces that contained the exact phrase “tips from former smokers” or all the words “government”, “campaign”, and “smoker”, or that included “CDC” in combination with “Tips”, “smoker”, “smoking”, or “tobacco.” To ensure that all relevant pieces were gathered, we repeated the searches on media outlet websites where searchable archives were available. This repeated search did not reveal any additional stories, suggesting that our search strategy was comprehensive for relevant content on the selected sites.

Our initial search strategy yielded 46 online media pieces; we reviewed each piece to confirm its relevance to the CDC Tips campaign. We excluded articles that included our keywords but did not mention Tips, for example, several that discussed state anti-smoking campaigns or CDC budget issues. We coded 36 pieces as relevant to the Tips campaign, defined by inclusion of at least a mention of the campaign, and 27 pieces as primarily focusing on Tips, which is reflected in story headlines that refer to the campaign. These 27 pieces were the focus of our final analysis, and each story was coded for publication date, presence of multimedia content (videos, pictures), and social media engagement data (shares, likes, tweets) (see Multimedia Appendix 1). We also coded whether pieces were posted to blogs or news sections of websites. The text of each piece was then subject to a basic thematic analysis of key content and messages. This basic thematic story coding was conducted by a single researcher (KS) using an open coding process that allowed for ongoing identification of emerging themes.

For media pieces where reader commenting was enabled (n=21), we manually compiled all comments in a Microsoft Access database. We coded comments that began new threads as “primary comments” (N=4040) and comments that responded to existing threads as “replies” (N=5507). We linked “reply” comments to the associated primary comment in the database. For each story, we calculated the reply rate, defined as the average number of replies generated by each primary comment.

In our initial review of the data, we noted that reply comments were often ambiguous without context from the threads from which they were generated (eg, “That's hot”, “I agree!!”, “Dana get a clue...”). We therefore focused our analysis on primary comments, which typically responded in some way to the media story. The volume of comments was such that we needed to create a sampling strategy to facilitate further coding of comments. We coded a simple random sample of primary comments across all media pieces (1370/4040, 33.91%) for two characteristics: (1) whether the comment mentioned the CDC Tips campaign, either by name or by reference to its cost, medium, or content, and (2) the comment’s valence with regard to Tips, or to governmental tobacco control efforts (where there was no specific mention of the campaign). Valence was coded as supportive (of tobacco control), oppositional, or unclear/irrelevant. A team of 5 trained coders analyzed the comments. Each comment was coded by 2 coders, and discrepancies were adjudicated by a third coder. To assess interrater reliability, Cohen’s kappa was calculated for all codes (Tips mention, .80; No Tips mention, .78; Support, .74; Opposition, .70; Unclear/Irrelevant, .45). For each article with comments, we took the first 20 primary comments (or all primary comments if <20) and performed open content coding to characterize the major justifications provided for support or opposition of the campaign. The first 20 comments were selected on the basis that the earliest comments are often displayed alongside articles and are most likely to be viewed and engaged with by readers.

The coding team then independently reviewed the comments to identify major themes. The themes then were discussed among all coders to aggregate overlapping codes and reach consensus on final categories. We conducted analyses of overall reader engagement (ie, Facebook likes, comments, tweets, shares), comment valence, and frequency of Tips campaign mentions in comments. We also made comparisons across media pieces as well as across websites.

## Results

### Online Blog and News Coverage of the Campaign

Our search of 5 online news sites and 9 policy blogs revealed 36 media pieces that mentioned the CDC’s “Tips from Former Smokers” campaign in the months following the campaign’s release. All the blog and news outlets we searched included at least one piece mentioning Tips with the exception of the National Review and RedState. Collected pieces had posting dates between March 14 and June 16, 2012, with the majority of stories accompanying campaign launch in March. Of these pieces, 23 were news articles, 11 were blog postings, and 2 were video or image reels (see Multimedia Appendix 1). Nine pieces mentioned but were not primarily focused on the Tips campaign, for example, two stories from the Wall Street Journal that summarized a number of news items (“AM Vitals”) and one from Huffington Post that mentioned Tips in the context of racial disparities in smoking rates (“Smoking Rates Increase With Perceived Racial Discrimination, Study Says”). The nine “non-Tips focused” pieces are included in Multimedia Appendix 2, but we did not analyze the public/audience engagement they generated.

Most articles (both those posted on news as well as blog sites) shared considerable content, suggesting that they were closely based on content from a press release or from a single wire (eg, Associated Press) story. Stories routinely reported on the fact that the Tips campaign is the largest ever federal program of its kind, as well as the cost of the campaign (US $54 million), the need for the campaign due to the stall in reduction of adult smoking rate (at around 20%), and the number of smokers (50,000) who might be expected to quit smoking after seeing the ads. Calling upon a wide variety of terminology, the news and blog pieces universally included a reference to the graphic nature of the ads (gruesome, difficult to watch, powerful, hard hitting, grisly, emotional, brutally honest, harsh). The actual ads were often either embedded in the story or described in detail in the text.

Assessment of the presentation of the campaign goals revealed some differences in coverage. Several stories focused on the ads’ potential to prompt quit attempts, whereas others identified their potential to prevent youth initiation. Stories typically related the ads to a desire to “shock”, “jolt”, or “scare” the audience, and act as a “wake-up call”. In contrast, few stories highlighted the potential for the ads to “educate”. The campaign was often presented in the context of other tobacco control policies whose impact has been limited or now seems to have stalled; tobacco taxes and smoking bans were specifically mentioned in this regard. The framing of campaign cost also differed somewhat between stories; some highlighted the extreme expense of the campaign while others contrasted the US $54 million spent by the federal government with the tobacco companies’ US $10 billion marketing budget.

Context for campaign coverage was provided by inclusion of quotes from government officials (namely, Kathleen Sebelius from US Health and Human Services and Thomas Frieden from the Centers for Disease Control and Prevention), as well as representatives of public health agencies and tobacco control organizations (John Seffrin from the American Cancer Society and Matthew Myers from Campaign for Tobacco Free Kids). A few stories mentioned that Philip Morris declined to officially comment, and one story provided a quote from an RJ Reynolds spokesperson comparing the ad campaign to the issue of graphic warnings on cigarette packs.

### Public Engagement

Engagement with online coverage of the CDC Tips campaign was tabulated from audience interaction data provided alongside the stories, although not all forms of engagement were reported by all outlets (see Multimedia Appendix 1). Across the 27 stories primarily focused on Tips, the most common form of engagement was commenting on the host site, with 9547 comments collected from the 21 pieces where commenting was enabled. Of these comments, 4040 began new threads, with the remainder responding to existing threads (average reply rate = 1.3). “Liking” stories on Facebook was the second most common form of engagement, with a total of 8891 likes reported for 18 stories where data were available. In total, 22 stories reported data related to Twitter for a total of 1027 tweets of story URLs; 10 stories reported a total of 505 shares of story URLs on Facebook.

**Figure 1:**
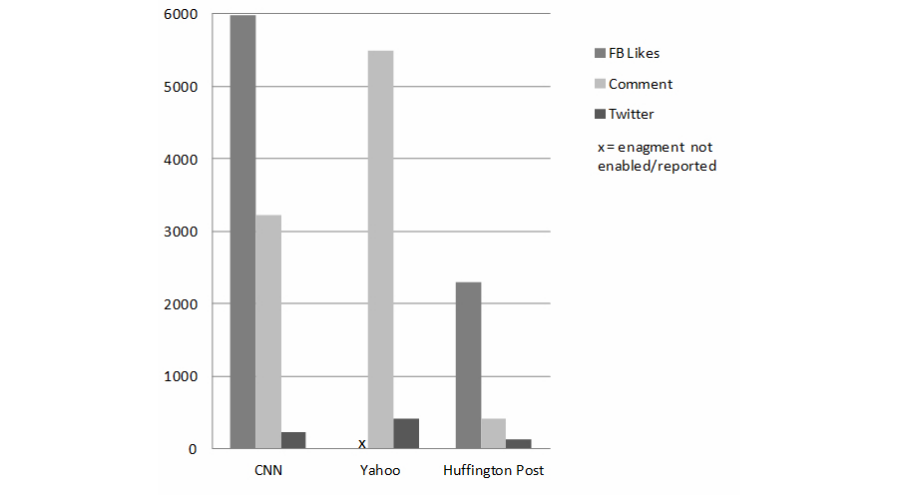
Level and mode of reader engagement with Tips news coverage on high engagement websites.

**Figure 2:**
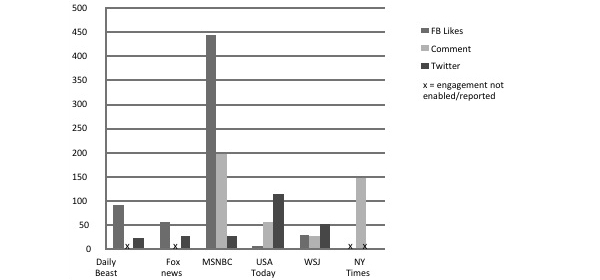
Level and mode of reader engagement with Tips news coverage on low engagement websites.

Mode and level of engagement varied considerably by story (see Multimedia Appendix 1) and by website (see Figures 1 and 2); CNN, Yahoo, and the Huffington Post were associated with considerably more engagement than other outlets. On CNN, Facebook likes were the most common form of engagement (n=5974), followed by commenting (n=3221) and tweets (n=237). On Yahoo, where Facebook likes were not enabled, there were a total of 5493 comments and 418 tweets. The Daily Beast and the Huffington Post both enabled Facebook shares, but only the Huffington Post was associated with a sizeable number of shares (n=484). The six Huffington Post stories were also associated with 2290 Facebook likes, 406 comments, and 133 tweets. Although the New York Times stories allowed sharing links through Facebook and Twitter, the number of shares was not reported. Politico, RedState, and the National Review did not include Tips-focused stories, and the LA Times coverage garnered a negligible level of engagement; these outlets are not represented in Figure 2.

### Comment Characteristics

**Figure 3:**
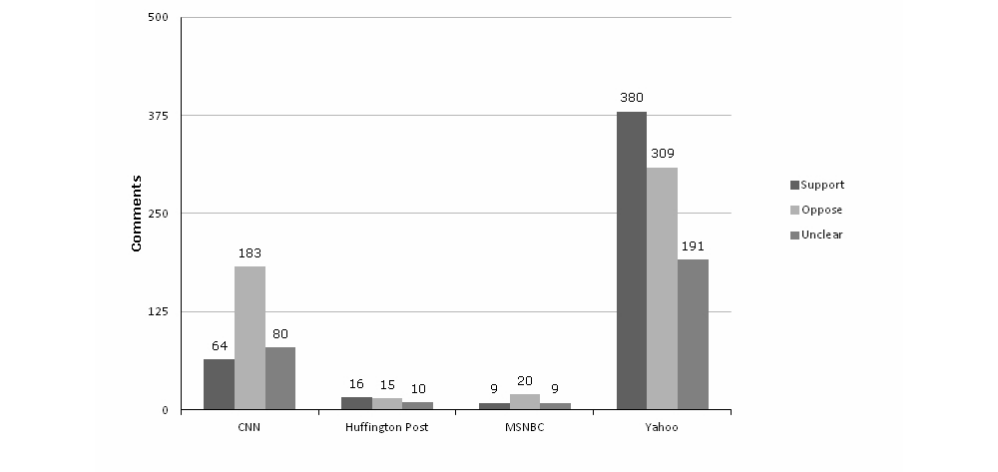
Valence of primary comments with regard to tobacco control, by website.

**Figure 4:**
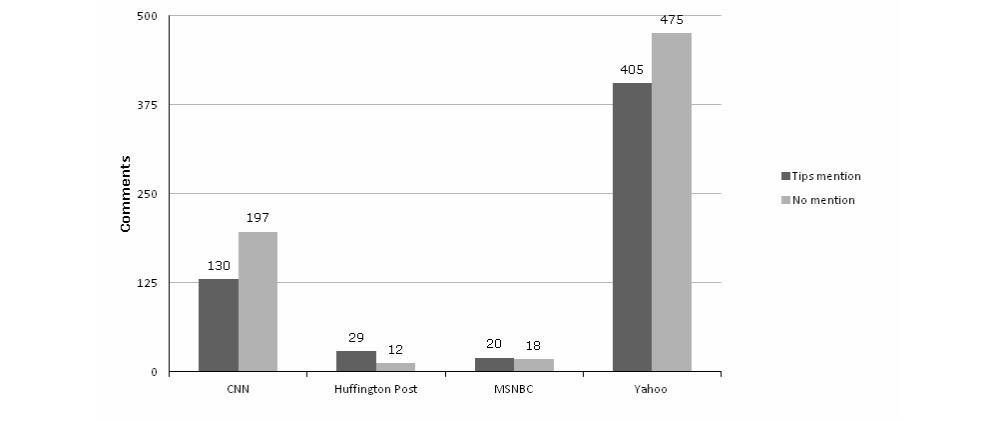
Comments with and without mentions of the CDC campaign, by website.

Figures 3 and 4 depict comment valence and frequency of campaign mentions in the sample of primary comments from four outlets, selected on the basis that each had a high number of public comments posted across multiple stories. The valence of comments varied by outlet/site, with the greatest proportion of supportive comments found on Yahoo (43.2%, 380/880) and the lowest on CNN (19.6%, 64/327) and MSNBC (24%, 9/38). The proportion of “on topic” comments mentioning the Tips campaign was highest on Huffington Post (71%, 29/41) and MSNBC (53%, 20/38) and lowest on CNN (39.8%, 130/327). Only one New York Times story allowed comments to be posted, and this piece was unusual in that it was a learning blog where school children responded to a prompt about the potential efficacy of the Tips campaign. The school children’s comments were largely formulaic and supportive, making this distinct from other comments sections.

We generated a thematic coding frame for comments by a process of open coding the first 20 primary comments for each story (n=270) (see Table 2). Below, we outline four predominant criticisms of the campaign within the comments that were coded as oppositional to the campaign as well as primary themes we identified within supportive comments. Among oppositional comments, we extracted themes of questioning: (1) the appropriateness of federal government’s involvement in influencing individuals’ health behaviors, (2) the allocation of funds and efforts to tobacco issues specifically, (3) the evidence upon which anti-smoking arguments are based, or (4) the effectiveness of campaigns. Comments supporting the campaign less frequently made an explicit case for the campaign, likely reflecting that most news/blog stories had already provided the campaign’s rationale. These comments instead tended to (1) disclose personal stories related to smoking, (2) express support for quitting or disapproval of smoking, and (3) convey reactions to the campaign.

Among oppositional comments, arguments regarding message appropriateness typically depicted governmental health promotion campaigns as a threat to individual liberties, often tying programs to the expanding role of government (eg, “...It used to be that, when a person was an adult, then he/she was in charge of his/her own actions. Now the Nanny State wants to control our actions...”). Arguments regarding allocation questioned why anti-smoking efforts were deemed more worthy of governmental attention than other health or policy issues such as other drugs or obesity (eg, “And just WHEN will we be seeing the same type anti-alcohol use billboards???”). Others questioned the evidence linking smoking to serious health outcomes (eg, “...everyone I have known to get cancer never smoked a cigarette in their lives...”) or questioned the efficacy of the ads (eg, “Everyone in the USA has known that smoking is hazardous to your health since the 1960s at least”). In contrast to oppositional comments, which typically developed explicit arguments against the campaign, supportive comments tended to endorse the campaign indirectly, often through personal anecdotes and reactions. Many commenters shared detailed narratives about their own or others’ experiences with smoking, smoking-related diseases, and with quitting. Others expressed the importance of not smoking, but varied greatly in their tone and civility; some offered support and encouragement to those attempting to quit while others expressed disdain for smoking. Finally, many responded to the content of the campaign, noting its personal impact or potential effectiveness.

**Table 2:** Major themes within oppositional and supportive comments.

Themes		Comments
**Oppositional comment themes**		
	**Appropriateness (role of government)**	
		The bottom line is that smoking is legal. If it is legal, then leave people alone. If people choose to ignore the health risks, then that is their business. Non-smokers should stop trying to control the behavior of others- smokers are no longer allowed to smoke indoors, near doorways, in bars, in public parks, etc. It used to be that, when a person was an adult, then he/she was in charge of his/her own actions. Now the Nanny State wants to control our actions. Liberty, do we even remember what the word means?
		What is really scary is the government interfering with peoples lives I read a book on this It was called The Communist Manifesto by Karl Marx
		People smoking is not the govt's business. if people smoke and get sick that is their problem and they can pay for the consequences.
		More big government. Why do we have to pay for this. Get the government out of our every day life,
	**Allocation**	
		I think diabetes and obesity are a much bigger and more fatal problem right now. Smoking can cause weight loss :-)
		And just WHEN will we be seeing the same type anti-alcohol use billboards??? Alcohol kills more innocent people than tobacco and drugs COMBINED!!!
		this is all fine and dandy, when are they going to show cancer stricken patients dying in their last days from skin cancer from to much time in tanning booths
		What marketing effort is the CDC doing to reduce the damage alcohol is doing to our society?
	**Evidence**	
		I know people who have smoked 50+ years and none of this stuff has ever happened to them. And they are still very healthy people! These commercials are a tad dramatic.
		How odd that the number of smokers has decreased (big-time), but people still get cancer (big-time). Matter of fact, everyone I have known to get cancer never smoked a cigarette in their lives. When are people going to figure out that we are going to die, no matter what we do?
		And how many smokers die of just old age?
		Buerger's Disease is very rare in the US and is more common in the middle and far east. I wish they would stop trying to buffalo people!
	**Effectiveness**	
		Everyone in the USA has known that smoking is hazardous to your health since the 1960s at least.
		People are not going to stop smoking just because of commercials, pictures, or warning labels. Even watching a family member die due to lung disease or cancer doesn't seem to convince people smoking is bad.
		If all this is aimed at keeping young people from smoking it isn't going to work. Young people just don't have the ability to care about what might happen to them when they are old (30 is old to them). At least have the good sense to show what it does to their teeth and skin. Something that they care about now. Now is all young people care about.
		Only the person that smokes, can make the decision to quit..NONE ELSE, can convince them to quit, NOT ADS,,NOT Family or friends or strangers..ONLY the FEAR in their own souls can make them quit!!!
**Supportive comment themes**		
	**Personal stories**	
		I had my last cigarette during my first heart attack in 1991. Gained a bunch of weight but I'm still kickin' in 2012.
		My mother died recently of congestive heart failure brought on by over 60 years of smoking. When My wife and I started cleaning her house for sale. It took over 4 weeks to just clean the tar and nicotine from the walls, ceilings, appliances, etc. Still didn't get it all. Once white curtains were dark yellow/brown. Her lungs had to look the same way. Bless you all.
		My dad has stopped smoking after smoking for 30+ years and I could not be any happier that he has.
	**Support for quitting/anti-tobacco**	
		Good luck to anyone who is trying to stop wish you nothing but the best!
		People stop Smoking !
		I can't believe in this day and age people still smoke....
	**Reactions to ad content**	
		I just saw the commercial with Terrie yesterday....I am still haunted. The scary and sad thing is quitting is so very hard and you never think things like that will happen to YOU! This PSA was probably one of the "best" I have ever seen.....I hope it helps a lot of people.
		These commercials are very effective. I quit over ten years ago, but if I had not, these new anti-smoking commercial would definitely make me want to.
		Very powerful! Thumbs up!

Table 3 depicts the overall levels of supportive and oppositional comments across the 27 media pieces that focused on the campaign. We excluded 297 comments from analysis on the basis that their valence was coded as unclear or irrelevant, leaving 1073 comments. Overall, 555 comments (51.72%) were coded as oppositional while 518 (48.28%) were coded as supportive of tobacco control. The fraction of oppositional comments was higher among those that mentioned Tips specifically (57.6%, 316/548), for stories posted to news sections of websites (73.6%, 290/394), and for stories that did not include videos from the Tips campaign (65.6%, 242/369). Reply rates were higher for comments with oppositional valence (1.6 vs 1.3 replies per comment). Reply rates were also higher for comments that did not mention the campaign specifically (1.7 vs 1.3 replies per comment).

**Table 3:** Characteristics of supportive and oppositional primary comments across Tips-focused stories (N=1073).

Characteristics	Opposition, n/N (%)	Support, n/N (%)
Total	555/1073 (51.72)	518/1073 (48.28)
Reply rate	1.6	1.3
Tips mention	316/548 (57.6)	232/548 (42.3)
No mention	239/525 (45.5)	286/525 (54.5)
Blog	265/679 (39.0)	414/679 (61.0)
News	290/394 (73.6)	104/394 (26.4)
Includes Tips video	313/704 (44.5)	391/704 (55.5)
No Tips video	242/369 (65.6)	127/369 (34.4)

## Discussion

### Principal Findings

We examined online earned media generated by the 2012 CDC Tips From Former Smokers campaign by compiling a diverse sample of leading online news outlets and blogs. We sought to summarize coverage of the campaign as well as indicators of public engagement. The volume of earned media coverage generated about Tips was fairly limited; we identified 36 pieces that mentioned the campaign, of which 27 were focused on Tips. Across our sample of 14 leading online news sources and blogs, most coverage clustered closely within a day of campaign launch. Our content analysis indicated that the news and blog pieces did not deviate greatly from the campaign press release. Media coverage of the campaign (including blog postings) generally included a set of core content with many pieces using visuals (photos or video) seemingly to provide exposure to or understanding of the ads’ graphic nature. The nature of the coverage did not suggest deep engagement with the issue on the part of journalists or bloggers. Earned media is sometimes seen as a way to develop understanding of an issue beyond what can be achieved in a 30-second or 60-second ad, but the news and blog pieces themselves provided little evidence of such development.

While the extent of earned media coverage about Tips may have been limited, even this small set of articles and postings generated notable audience engagement in the form of social media shares and comments posted on news/blog sites. The stories yielded over 9000 comments and almost 9000 Facebook likes, in addition to diffusion and dissemination of content via a variety of other social media channels. Such levels of audience engagement may indicate that the campaign and the issues it raises are salient to a sizable number of people [34]. The social media and comment data available adjacent to a story may also serve to indicate story importance (agenda-setting) to other audience members, with a potential cyclical effect. No directly comparable studies are available with which to compare the engagement levels found in this research. However, although drawn from different sources and related to different topics, two prior studies did suggest a much lower level of commenting on average than was found in this study [25,35]. Our research may provide a baseline that future studies can build upon to analyze audience engagement.

Levels of engagement varied considerably between sources, ranging from fewer than 100 shares/likes/comments to several thousand for a single piece (on the CNN site). Notably, not all sites facilitated user engagement in the same way; FoxNews and USA Today, for example, enabled comments only on a subset of the stories they posted. If audience engagement is to become part of how we conceptualize earned media, it is important to better understand why some sites generate far more active engagement than others, and the rationale behind decisions by some sites not to facilitate certain sharing or commenting mechanisms. Such insight could lead to targeted efforts to generate interest on the part of key journalists/writers who serve as gatekeepers to extensive social networks for information diffusion.

The review of user-submitted public comments revealed that they did not universally reference the campaign directly and many had unclear valence. Only about half of comments explicitly referenced the Tips campaign, although most others did include more general discussion of the role of government, the value of government-funded health campaigns, tobacco control policy, and health effects of tobacco use (see Multimedia Appendix 1). In some cases, no clear link to tobacco control (very broadly defined) could be discerned. Furthermore, we could not discern the valence of 297 of 1370 coded comments (21.68%) either because they were ambiguous or off-topic. Some of these off-topic comments might be seen as “trolling” or “spam” but, in any case, it was difficult to assess any relevance to the campaign other than the presence of negative noise [14].

There was an almost even split between the valence of comments across all Tips-focused pieces between those that were oppositional to a public health position (51.72%, 555/1073) and those that presented a supportive stance (48.28%, 518/1073). Thus, these data suggest that raw counts of audience engagement would be of limited utility to assess the likely effectiveness of earned media in supporting a campaign; by no means did all comments indicate support for the campaign or the issues it touched upon (including acknowledgement of the harmful effects of tobacco). The fact that comments explicitly referencing the Tips campaign were more likely to espouse an oppositional perspective suggests that the campaign itself may be more challenging than tobacco control more broadly conceptualized, or perhaps that oppositional comments reflected a more considered effort at argumentation. It is possible that the Tips campaign may be effectively pushing against oppositional commenters’ opinions and knowledge in such a way as to move public opinion over time. Alternatively, recent research suggests that some common public health messaging approaches not only fail to influence target populations as intended but may in fact strengthen opponents’ resolve [36]. The variety of online public forums may provide an efficient avenue for such parties to quickly register their opposition and to advance counter-arguments. Accordingly, when crafting press releases, it is important to consider the arguments that will inevitably surface in relation to tobacco and tobacco control (smoking as an issue of individual liberty, for example) and address these in relation to any specific story. This point also speaks to the importance of agenda-setting and framing of messages about the campaign; for example, a story may or may not compare levels of campaign advertising spending to the amount spent on tobacco advertising and may or may not address the issue of individual liberties, and so forth.

Our analysis of the relationship between website and story features and levels of supportive and oppositional engagement revealed both surprising and unsurprising findings. Consistent with our expectations, we found that stories that included video from the Tips campaign were associated with more supportive commentary, suggesting that exposure to the ads themselves may have achieved positive influence. In contrast, we were surprised to find that oppositional comments were more prevalent in response to news stories as compared with blog posts. Further comparison of the nature of commentary in response to blogs (which are explicitly opinionated) versus news pieces is warranted to fully understand the value of each to shaping public discourse. There were also differences in comment valence by website, with comments on Yahoo more likely to be supportive than those on MSNBC or CNN. Such differences are difficult to explain from this analysis, and the findings do not align easily with the political perspective espoused by these various news sources. Perhaps these findings point to more spam/troll activity on some sites than on others.

Developing effective methods for tracking news media and consumer responses to public health campaigns is critical for public health efforts. An analysis of the interactive online environment provides an opportunity for the public health community to monitor sentiment in real time, identify the most productive platforms and pathways for information sharing, and anticipate and address points of resistance. This paper is focused on earned media as it manifests in online outlets.

### Limitations

We faced several limitations in conducting this research. First, because we limited our analysis to a subset of news and blog websites, we are unable to provide a comprehensive view of online earned media related to the Tips campaign. In measuring public engagement, we were constrained by the fact that the sample websites did not have uniform ways of reporting engagement metrics, and some metrics were not reported at all by some sites. Although the New York Times stories we collected were likely associated with sharing via social media such as Twitter, the number of shares was not reported alongside these stories at the time of data collection. We also did not characterize login procedures or standards of anonymity among the websites we examined, although these features may affect decisions of whether and how readers respond [26,37,38]. Further, although we characterized the major themes appearing in campaign coverage, we did not examine whether different ways of framing the campaign affected the level and type of audience engagement. Given the websites’ varying standards of reporting commenters’ names and the possibility that a commenter might post under multiple names or accounts, we also could not characterize the distribution of total comments across unique commenters. Our content analysis of commentary was limited by our difficulty in interpreting some comments, especially given their variety and informality of language and potential for sarcasm, perhaps contributing to our marginal kappa score for the unclear/irrelevant valence category [39]. Replies to existing comment threads were particularly ambiguous, and thus we excluded them entirely. Furthermore, there is some difficulty in interpreting the levels of support and opposition we identified since it is not known to what extent commenters represent the general population or even each outlet's readership. We also do not know whether commentary reflected any organized efforts to promote an agenda with regard to tobacco control or the Tips campaign, although we did not detect evidence for this such as repeated blocks of text across comments. Finally, the content and valence of comments are likely to influence how stories are interpreted, but it is further unknown what fraction of readers consults comments, and this is likely to vary by outlet.

### Conclusions

The large scale and graphic nature of the Tips campaign gave it potential to obtain important levels of earned media on both traditional and online platforms. While the campaign may have received limited coverage on either online news or blog sources, earned media that did cover Tips generated significant engagement among online audiences. To extend their reach and impact, future tobacco control campaigns should include advocacy efforts to capture the attention of a wider set of journalists and opinion leaders in order to build earned media and facilitate productive public engagement.

## Multimedia Appendix 1

Supplemental table showing online news and policy blog coverage of CDC’s “Tips From Former Smokers” Campaign. Stories primarily focused on CDC’s Tips From Former Smokers Campaign.

## Multimedia Appendix 2

Supplemental table showing stories not primarily focused on CDC’s Tips From Former Smokers Campaign.

## Abbreviations

CDC: Centers for Disease Control and Prevention
PSA: public service announcement
Tips: Tips From Former Smokers campaign

## References

[1] Centers for Disease Control and Prevention Tips From Former Smokers.

[2] MacKenzie R, Chapman S (2012). Generating news media interest in tobacco control: Challenges in an advanced policy environment. Health Promot J Austr.

[3] Wakefield MA, Brennan E, Durkin SJ, McLeod K, Smith KC (2011). Still a burning issue: Trends in the volume, content and population reach of newspaper coverage about tobacco issues. Crit Public Health.

[4] Pederson LL, Nelson DE, Babb S, London J, Promoff G, Pechacek T (2012). News media outreach and newspaper coverage of tobacco control. Health Promot Pract.

[5] Balbach ED, Glantz SA (1998). Tobacco control advocates must demand high-quality media campaigns: the California experience. Tob Control.

[6] (2012). Pew Res Cent People Press.

[7] Smith KC, Niederdeppe J, Blake KD, Cappella JN (2013). Advancing cancer control research in an emerging news media environment. J Natl Cancer Inst Monogr.

[8] Emery SL, Vera L, Huang J, Szczypka G (2014). Wanna know about vaping? Patterns of message exposure, seeking and sharing information about E-cigarettes across media platforms. Tob Control.

[9] Tremayne M, Weiss AS, Alves RC (2007). From product to service: The diffusion of dynamic content in online newspapers. Journal Mass Commun Q.

[10] Stroud NJ, Scacco J, Curry A (2013). Engaging News Project.

[11] Hall Jamieson K, Cappella JN (2010). Echo chamber: Rush Limbaugh and the conservative media establishment. Reprint edition.

[12] Johnson TJ, Kaye BK (2013). The dark side of the boon? Credibility, selective exposure and the proliferation of online sources of political information. Comput Hum Behav.

[13] Johnson TJ, Kaye BK (1998). Cruising is believing?: Comparing Internet and traditional sources on media credibility measures. Journal Mass Commun Q.

[14] Anderson AA, Brossard D, Scheufele DA, Xenos MA, Ladwig P (2013). The "Nasty Effect": Online incivility and risk perceptions of emerging technologies. J Comput-Mediat Commun.

[15] Shi R, Messaris P, Cappella J (2104). Effects of online comments on smokers’ perception of antismoking public service announcements. J Comput-Mediat Commun.

[16] Scheufele DA, Tewksbury D (2007). Framing, agenda setting, and priming: The evolution of three media effects models. J Commun.

[17] Entman RM (1993). Framing: Toward clarification of a fractured paradigm. J Commun.

[18] Gollust SE, Lantz PM, Ubel PA (2009). The polarizing effect of news media messages about the social determinants of health. Am J Public Health.

[19] Moreland-Russell S, Harris JK, Israel K, Schell S, Mohr A (2012). "Anti-smoking data are exaggerated" versus "the data are clear and indisputable": examining letters to the editor about tobacco. J Health Commun.

[20] Harris JK, Cohen EL, Wyrwich KW, Luke DA (2011). Differences in smokers and nonsmokers’ assessments of an educational campaign about tobacco use. Health Educ Behav.

[21] Harris JK, Shelton SC, Moreland-Russell S, Luke DA (2010). Tobacco coverage in print media: The use of timing and themes by tobacco control supporters and opposition before a failed tobacco tax initiative. Tob Control.

[22] Wackowski OA, Lewis MJ, Hrywna M (2011). Banning smoking in New Jersey casinos—A content analysis of the debate in print media. Subst Use Misuse.

[23] Fahy D, Trench B, Clancy L (2009). Communicating contentious health policy: Lessons from Ireland's workplace smoking ban. Health Promot Pract.

[24] Neuman WR, Guggenheim L, Jang SM, Bae SY (2014). The dynamics of public attention: Agenda-setting theory meets big data. J Commun.

[25] Coe K, Kenski K, Rains SA (2014). Online and uncivil? Patterns and determinants of incivility in newspaper website comments. J Commun.

[26] Moore MJ, Nakano T, Enomoto A, Suda T (2012). Anonymity and roles associated with aggressive posts in an online forum. Comput Hum Behav.

[27] Santana A (2012). University of Oregon.

[28] Cho D, Kim S, Acquisti A (2012). Empirical analysis of online anonymity and user behaviors: the impact of real name policy. System Science (HICSS).

[29] Hlavach L, Freivogel WH (2011). Ethical implications of anonymous comments posted to online news stories. J Mass Media Ethics.

[30] Zhuo J (2010). NY Times Internet.

[31] Salathé M, Vu DQ, Khandelwal S, Hunter DR (2013). The dynamics of health behavior sentiments on a large online social network. EPJ Data Sci.

[32] (2012). Top US web brands and news websites.

[33] (2012). ComScore Media Metrix Ranks Top 50 US Web Properties for August 2012.

[34] Henrich N, Holmes B (2011). What the public was saying about the H1N1 vaccine: perceptions and issues discussed in on-line comments during the 2009 H1N1 pandemic. PLoS One.

[35] Yano T, Smith NA (2010). What's worthy of comment? Content and comment volume in political blogs. Proceedings of the Fourth International AAAI Conference on Weblogs and Social Media.

[36] Nyhan B, Reifler J, Richey S, Freed GL (2014). Effective messages in vaccine promotion: A randomized trial. Pediatrics.

[37] Broekhuizen T, Hoffmann A (2012). Interactivity perceptions and online newspaper preference. J Interact Advert Internet.

[38] Velasquez A (2012). Social media and online political discussion: The effect of cues and informational cascades on participation in online political communities. New Media & Society.

[39] Schneider J, Davis B, Wyner A (2012). Dimensions of argumentation in social media. Knowledge Engineering and Knowledge Management.

