# Supplementary material for: Earned Media and Public Engagement With CDC’s "Tips From Former Smokers" Campaign: An Analysis of Online News and Blog Coverage
Source: J Med Internet Res. 2015 Jan 20;17(1):e12. doi: 10.2196/jmir.3645 (PMC4319092; doi:10.2196/jmir.3645)
Supplement: Supplementary file 1 [file jmir_v17i1e12_app1.pdf]

Multimedia Appendix 1. Online news and policy blog coverage of CDC's "Tips From Former Smokers" Campaign
Stories primarily focused on CDC's Tips From Former Smokers Campaign.

Website	News vs. Blog	Story Date	Story Headline	Tips video	FB Likes	FB Shares	Tweets	Other (G+, email, InShare, unspecified)	Total Comments	Primary Comments (N)	Reply Rate	Max Replies
CNN	news	3/16/2012	CDC unveils graphic ads to combat smoking	no	4600	--	146	39	2307	689	2.3	29
	news	1/9/2013	New anti-smoking ads a smart move	yes	174	--	16	6	752	244	2.1	18
	news	3/16/2012	Anti-smoking symbol reveals 'worst moment'	yes	1200	--	75	7	162	61	1.7	10
Daily Beast	news	3/15/2012	Graphic Anti-Smoking Campaign Debuts	no	17	6	6	0	0	0	--	--
	blog	3/16/2012	Could Graphic New Anti Smoking Ads Do More Harm?	yes	73	15	16	0	0	0	--	--
Fox News	other	3/15/2012	Govt's Ad Campaign on Gruesome Effects of Smoking (Videos)	no	--	--	--	0	--	--	--	--
	news	3/15/2012	Ad campaign shows smoking's scary side	no	33	--	22	1	0	0	--	--

Website	News vs. Blog	Story Date	Story Headline	Tips video	FB Likes	FB Shares	Tweets	Other (G+, email, InShare, unspecified)	Total Comments	Primary Comments (N)	Reply Rate	Max Replies
	news	3/15/2012	CDC Launches Graphic Anti-Smoking Campaign	yes	24	--	3	27	0	0	--	--
Huffington Post	news	3/15/2012	CDC Anti-Smoking Ad Campaign Set To Launch	no	1379	317	36	31	341	86	3	17
	news	3/19/2012	Anti-Smoking Ads 2012: CDC Offers GRAPHIC Reasons To Quit Smoking	yes	828	133	40	67	39	21	0.9	4
	blog	4/23/2012	Do Anti-Tobacco Ads Work? Ask a 'Neural Focus Group'	no	62	24	49	4	19	12	0.6	6
	news	3/30/2012	Calls Double To Quit-Smoking Hotline After Anti-Cigarette Ads Debut	no	21	10	8	11	7	7	0	--
LA Times	news	3/19/2012	New ads tally smoking's toll	no	1	--	0	0	--	--	--	--
MSNBC	news	3/15/2012	Scary anti-smoking ad campaign launches	no	444	--	26	174	197	106	0.9	21

Website	News vs. Blog	Story Date	Story Headline	Tips video	FB Likes	FB Shares	Tweets	Other (G+, email, InShare, unspecified)	Total Comments	Primary Comments (N)	Reply Rate	Max Replies
	news	3/15/2012	Graphic new smoking campaign designed to shock	no	--	--	--	0	--	--	--	--
NY Times	blog	3/19/2012	Are Antismoking Ads Effective?	no	--	--	--	0	147	147	0	--
	news	3/15/2012	U.S. Backs Antismoking Ad Campaign	yes	--	--	--	0	--	--	--	--
USA Today	news	3/14/2012	CDC to launch graphic anti-smoking ads	no	0	--	35	1	30	13	1.3	7
	news	3/30/2012	CDC: Ads spark huge increase to quit smoking line	yes	6	--	79	0	26	14	0.9	2
	news	3/15/2012	CDC to launch anti-tobacco crusade	no	0	--	0	0	--	--	--	--
Wall Street Journal	news	3/15/2012	U.S. Unveils Antismoking Campaign	no	24	--	44	2	27	13	1.1	4
	other	3/15/2012	New Antismoking Ads (Slideshow)	no	5	--	8	0	--	--	--	--
Yahoo	blog	3/20/2012	Anti-smoking PSAs offer tips on life with a tracheotomy	yes	--	0	280	41	4916	2311	1.1	63

Website	News vs. Blog	Story Date	Story Headline	Tips video	FB Likes	FB Shares	Tweets	Other (G+, email, InShare, unspecified)	Total Comments	Primary Comments (N)	Reply Rate	Max Replies
	news	3/15/2012	CDC launching graphic anti-smoking ad campaign	no	--	0	116	19	560	305	0.8	12
	news	3/16/2012	The CDC's shocking new anti-smoking campaign: Will it work?	no	--	0	0	0	14	8	0.8	3
	news	6/21/2012	Anti-Smoking Ads Have Increased Quit Attempts: CDC	no	--	0	22	2	3	3	0	--
Washington Times	news	3/15/2012	CDC launching graphic anti-smoking ad campaign	no	--	--	--	0	0	0	--	--
			Stories reporting engagement		18	10	22	27	21	21		
			Total		8891	505	1027	432	9547	4040	1.3	
