# Supplementary material for: Earned Media and Public Engagement With CDC’s "Tips From Former Smokers" Campaign: An Analysis of Online News and Blog Coverage
Source: J Med Internet Res. 2015 Jan 20;17(1):e12. doi: 10.2196/jmir.3645 (PMC4319092; doi:10.2196/jmir.3645)
Supplement: Supplementary file 2 [file jmir_v17i1e12_app2.pdf]

Multimedia Appendix 2. Supplemental table showing stories *not* primarily focused on CDC's Tips From Former Smokers Campaign.

Website	News vs. Blog	Story Date	Story Headline	FB Likes	FB Shares	Tweets	Other	Total Comments	Primary Comments (N)	Reply Rate
Huffington Post	news	3/16/2012	Smoking Rates Increase With Perceived Racial Discrimination, Study Says	116	49	3	9	81	55	0.5
	blog	6/20/2012	Bad Breath: All Causes, Great and Small	186	140	119	7	1	1	0
Politico	blog	4/29/2012	5 prevention programs GOP hopes to target	56	--	58	0	9	9	0
	blog	3/15/2012	Politico Pulse: Dems hit Mitt on Planned Parenthood…	--	--	--	2	1	1	0
	blog	4/30/2012	Politico Pulse: Next move on Prevention Fund unclear…	--	--	--	2	0	0	--
WSJ	blog	3/20/2012	A.M. Vitals: Training the Body to Grow New Parts	3	--	1	0	4	4	0
	blog	3/15/2012	A.M. Vitals: Doctors Fear Squeeze as Reimbursement Schemes Evolve		--	1	0	2	2	0
	news	3/15/2012	U.S. WATCH: HEALTH	--	--	0	0	1	1	0
Washington Times	blog	3/24/2012	Graphic Cigarette Labels: A Step Backwards in Combating Nicotine Addiction	--	--	--	0	2	2	0
